# An Observational Study of the Progression of Patients’ Mental Health Symptoms Six Weeks Following Discharge From the Hospital

**DOI:** 10.1192/j.eurpsy.2024.1263

**Published:** 2024-08-27

**Authors:** W. Mao, R. Shalaby, E. Eboreime, E. Owusu, H. Elgendy, N. Shalaby, B. Agyapong, N. Nkire, A. Nichols, V. I. Agyapong

**Affiliations:** ^1^Psychiatry, University of Alberta, Edmonton; ^2^Psychiatry, Dalhousie University, Halifax; ^3^Queen Elizabeth II Hospital, Alberta Health Services, Grande Prarie, Canada

## Abstract

**Introduction:**

Transitioning from mental health inpatient care to community care is often a vulnerable time in the treatment process where additional risks and anxiety may arise.

**Objectives:**

The objective of this paper was to evaluate the progression of mental health symptoms in patients six weeks after their discharge from the hospital as the first phase of an ongoing innovative supportive program. In this study, factors that may contribute to the presence or absence of anxiety and depression symptoms, and the quality of life following a return to the community were examined. The results of this study provide evidence and baseline data for future phases of the project.

**Methods:**

An observational design was used in this study. We collected sociodemographic and clinical data using REDCap at discharge and six weeks later. Anxiety, depression, and well-being symptoms were assessed using the Generalized Anxiety Disorder (GAD-7) questionnaire, the Patient Health Questionnaire-9 (PHQ-9), and the World Health Organization-Five Well-Being Index (WHO-5) respectively. Descriptive, Chi-square, independent T-test, and multivariate regression analyses were conducted.

**Results:**

The survey was completed by 88 participants out of 144 (61.1% response rate). A statistically non-significant reduction in anxiety and depression symptoms was found six weeks after returning to the community based on the Chi-squared/Fisher exact test and independent t-test. As well, the mean anxiety and depression scores showed a non-significant marginal reduction after discharge compared to baseline. In the period following discharge, a non-significant increase in participants experiencing low well-being symptoms was observed, as well as a decline in the mean well-being scores. Based on logistic regression models, only baseline symptoms were significant predictors of symptoms six weeks after inpatient discharge.

**Image:**

**Figure:**
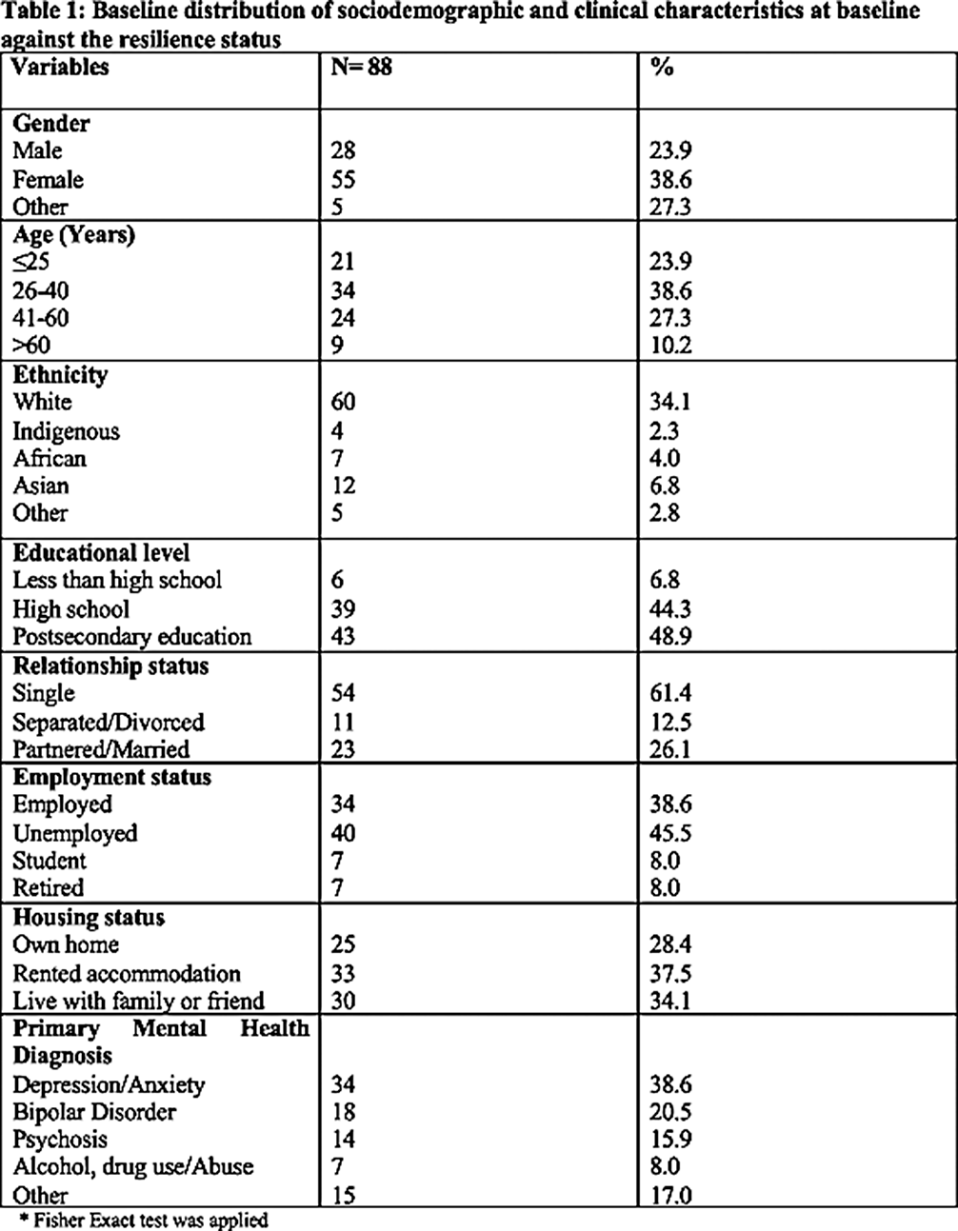

**Image 2:**

**Figure:**
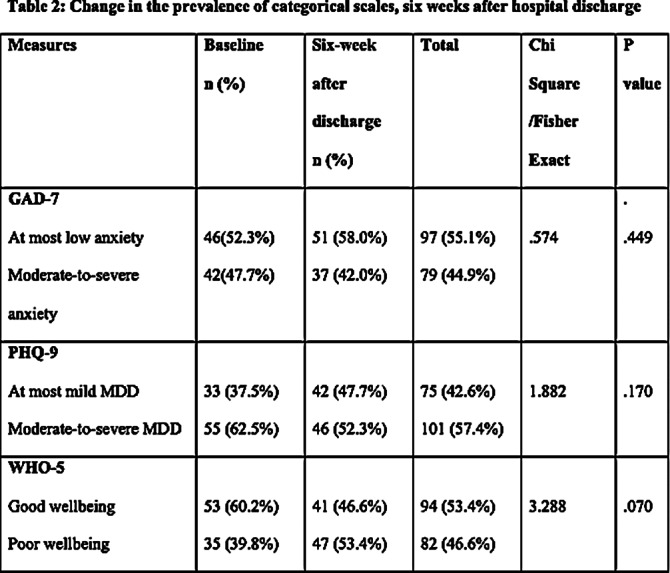

**Image 3:**

**Figure:**
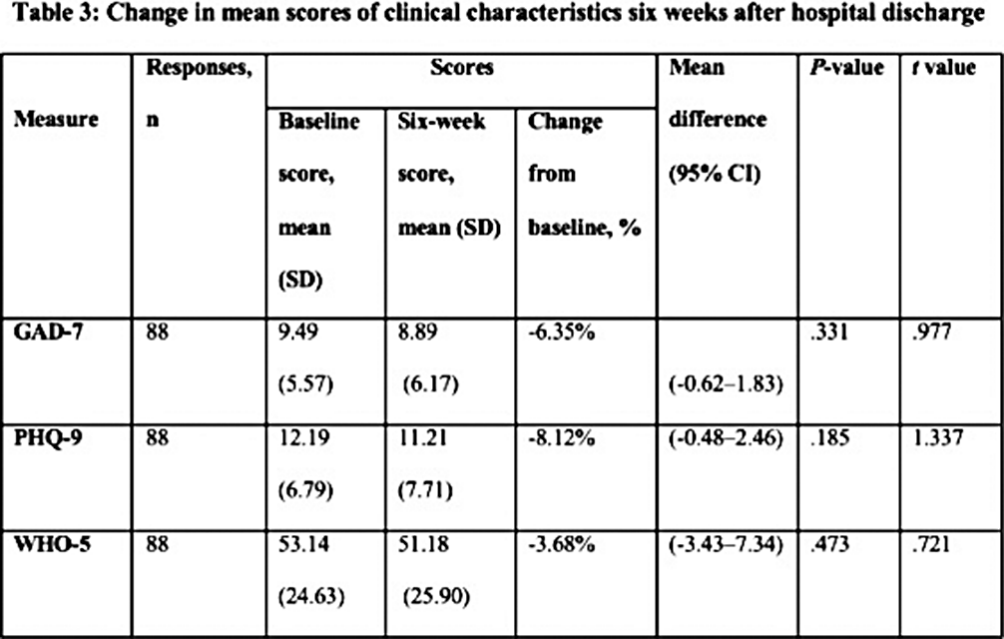

**Conclusions:**

In the short term following hospital discharge, no significant changes were observed in mental health conditions. A collaboration between researchers and policymakers is essential for the implementation and maintenance of effective interventions to support and maintain the mental health of patients following discharge.

**Disclosure of Interest:**

None Declared

